# Association between intra-arterial catheterization and mortality of acute heart failure patients without shock in ICU: A retrospective study

**DOI:** 10.1016/j.ahjo.2024.100432

**Published:** 2024-07-29

**Authors:** Yide Li, Yuan Zhu, Le Fu, Liang Luo, Yingfang She

**Affiliations:** a Department of Critical Care Medicine, The Seventh Affiliated Hospital, Sun Yat-Sen University, Shenzhen, China; b Neurology Medicine Center, The Seventh Affiliated Hospital, Sun Yat-Sen University, Shenzhen, China

**Keywords:** Intra-arterial catheters, Acute heart failure, Hemodynamically stable, Mortality, MIMIC IV database

## Abstract

**Background:**

Acute heart failure necessitates intensive care, and arterial catheterization is a commonly performed invasive procedure in the intensive care unit (ICU). We aimed to investigate the association between arterial catheterization and outcomes in acute heart failure patients without shock.

**Methods:**

We utilized MIMIC-IV database records for acute heart failure patients at Beth Israel Deaconess Medical Center from 2008 to 2019. Employing doubly robust estimation, we examined the relationship between arterial catheterization and outcomes, including 28-day, 90-day, in-hospital mortality, and ICU-free days within 28 days.

**Results:**

Of 6936 patients identified, 2078 met inclusion criteria; 347 underwent arterial catheterization during their ICU stay. We observed no significant difference in 28-day mortality (odds ratio [OR]: 0.61, 95 % confidence interval [CI]: 0.31–1.21, *P* = 0.155), though catheterization was associated with reduced in-hospital mortality (OR: 0.41, 95 % CI: 0.14–0.65, *P* = 0.02). No significant effects were observed on 90-day mortality or ICU-free days within 28 days.

**Conclusion:**

Our findings suggest that arterial catheterization is not associated with 28- and 90-day mortality rates in acute heart failure patients without shock but is linked to lower in-hospital mortality. Additional research and consensus are required to determine the appropriate utilization of arterial catheterization in patients.

## 1 Background

The global prevalence of heart failure, a chronic and incurable condition, is on the rise, significantly adding to the worldwide disease burden. As of 2017, it was estimated that over 64.3 million individuals globally suffered from heart failure, with a notable increase among the aging population, where it stands as the leading reason for hospital admissions (1,2). Acute heart failure often necessitates immediate medical attention in the event of rapid deterioration in signs and symptoms. Over 10 % of these patients require admission to the intensive care unit (ICU) (3,4).

Arterial catheterization is a more commonly performed invasive procedure in managing ICU patients, enabling precise monitoring across various acute conditions. Studies reveal its steady utilization in about 36 % of ICU cases from 2001 to 2008, underscoring its established role in critical care (5). Despite widespread application, the prognostic significance of arterial catheterization in hemodynamically stable patients remains contentious ([6], [7], [8], [9]). Concerns about its invasiveness and associated risks like infection and thrombosis (10,11) contrast with mixed evidence on its impact on mortality and healthcare costs in stable patients ([12], [13], [14], [15]). Concurrently, we lack comprehensive knowledge of the association between mortality and intra-arterial catheterization in acute heart failure patients without shock.

Therefore, we conducted a single-center, retrospective study to assess the impact of indwelling intra-arterial catheters on mortality in patients in acute heart failure patients without shock.

## 2 Methods

### 2.1 Design and ethical considerations

This single-center, retrospective study adhered to the guidelines outlined in the Strengthening the Reporting of Observational Studies in Epidemiology (STROBE) statement (16). This study was based on a high-quality deidentified dataset. The Institutional Review Boards at both Massachusetts Institute of Technology and the Beth Israel Deaconess Medical Center (BIDMC) approved the use of the data for research.

### 2.2 Study population

The Medical Information Mart for Intensive Care-IV (MIMIC-IV) database originates from the BIDMC and comprises data from their custom hospital-wide electronic health records and ICU-specific clinical information systems. The database includes patient data from the BIDMC emergency department and ICUs from 2008 to 2019. MIMIC-IV underwent data acquisition, preparation, and deidentification processes to ensure data usability and patient privacy (17). In this study, we utilized the latest version (2.2) of the database and performed data search and extraction using PostgreSQL v11.1(http://www.postgresql.org/).

We assessed the independent impact of intra-arterial catheters on mortality in acute heart failure patients without shock managed in the ICU. The inclusion criteria were as follows: (1) primary diagnosis of acute heart failure or related diagnoses and (2) patients aged ≥18 years. Exclusion criteria included: (1) patients with previous ICU admissions, (2) ICU length of stay <1 d, (3) patients who had intra-arterial catheters prior to ICU admission, and (4) patients who received vasoactive medications (norepinephrine, epinephrine, phenylephrine, vasopressin, dopamine) during their ICU stay. Exclusion of patients receiving vasoactive medications ensured the selection of hemodynamically stable individuals, as the use of such medications is a more precise indicator of shock than blood pressure readings alone.

The International Classification of Diseases (ICD) was used to identify relevant diagnoses related to acute heart failure. The specific ICD codes and their versions corresponding to the respective diagnoses are presented in Supplementary Table S1.

### 2.3 Data extraction

Patient demographic characteristics, vital signs, comorbidities, laboratory findings, intra-arterial placement, illness severity score, prognosis, length of ICU stay, and special treatment (activation of Continuous Renal Replacement Therapy [CRRT] or mechanical ventilation) within 24 h of admission to the ICU were extracted from the database. Laboratory results were extracted as the worst value, and vital signs were extracted as the mean value recorded within 24 h of admission to the ICU.

### 2.4 Primary and secondary outcome

The primary outcome was the 28-day mortality rate of the patients, while secondary outcomes encompassed the 90-day, in-hospital mortality rates, and the number of ICU-free days within 28 days.

### 2.5 Statistical analysis

We utilized a doubly robust estimation method to ascertain the independent relationships between intra-arterial catheters and the primary and secondary patient outcomes. This technique integrates a multivariate regression model with a propensity score model, allowing for the estimation of associations and causal impacts of catheter use on outcomes (18,19). Unlike traditional methods that require precise definitions in both outcome regression and propensity score models for unbiased results, the doubly robust estimator only needs one of these models to be correctly specified to produce an unbiased estimate.

To address potential biases, such as overfitting and selection bias in our regression analysis, we used Extreme Gradient Boosting (XGBoost), a sophisticated machine learning algorithm, for estimating propensity scores, combined with the doubly robust estimation approach. Although this method enhances the precision and reliability of our results, it's important to note that no method can entirely remove all biases present in retrospective studies. We then applied an inverse probability weighting (IPW) model to generate a weighted cohort, using the estimated propensity scores as weights. Subsequently, logistic regression was conducted on this weighted cohort to balance any remaining discrepancies. This regression adjusted for covariates showing imbalances in the propensity score model between patients with and without intra-arterial catheters. By integrating logistic regression with the IPW model, we bolstered the robustness and credibility of our analysis.

Continuous variables are presented as the mean and standard deviation (SD), whereas categorical variables are presented as numbers and percentages. The Mann–Whitney *U* test and Fisher's exact tests were used to compare continuous and categorical variables, respectively. For matched data, the paired *t*-test and McNemar's test were used to assess continuous and categorical data, respectively. All significance tests were two-sided, and a *P* value <0.05 was considered statistically significant. Statistical analysis was conducted using the R software (version 4.3.0; R Foundation for Statistical Computing, Vienna, Austria; https://www.r-project.org).

### 2.6 Sensitivity analysis

We performed various sensitivity analyses to assess the robustness of our findings and the impact of different association inference models. This included the use of three additional models: a doubly robust model adjusted for all covariates, a propensity score-based IPW model, and a propensity score-based patient-matching model. We compared the effect sizes and *P*-values obtained from each model to ensure the reliability of the conclusions.

## 3 Results

### 3.1 Study population

**Fig. 1:**
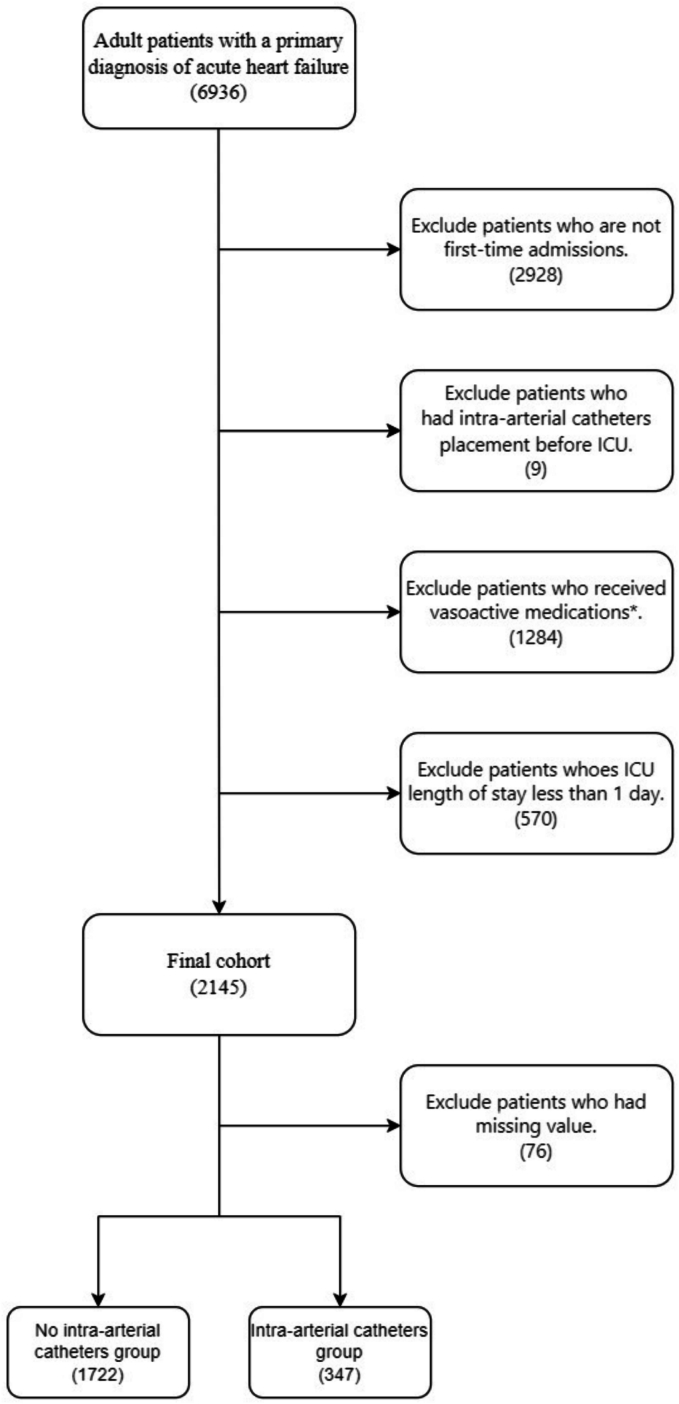
Flowchart outlining the cohort selection process.

A total of 6936 adult patients with a primary diagnosis of acute heart failure were identified in the MIMIC-IV v2.2 database. A cohort of 2145 eligible patients met the selection criteria. Among these, a small fraction of approximately 3.5 % (76/2145) had missing values. After removing patients with missing information to ensure a complete case analysis, the cohort was reduced to 2078 patients. Among the remainder of the cohort, 347 patients had intra-arterial catheters during their ICU stay, whereas 1722 patients did not (Fig. 1). Table 1 presents the baseline characteristics of the study cohort. The group with arterial catheters exhibited a younger age, higher mean arterial pressure, and an increased proportion of patients on mechanical ventilation.

### 3.2 Primary outcome

**Fig. 2:**
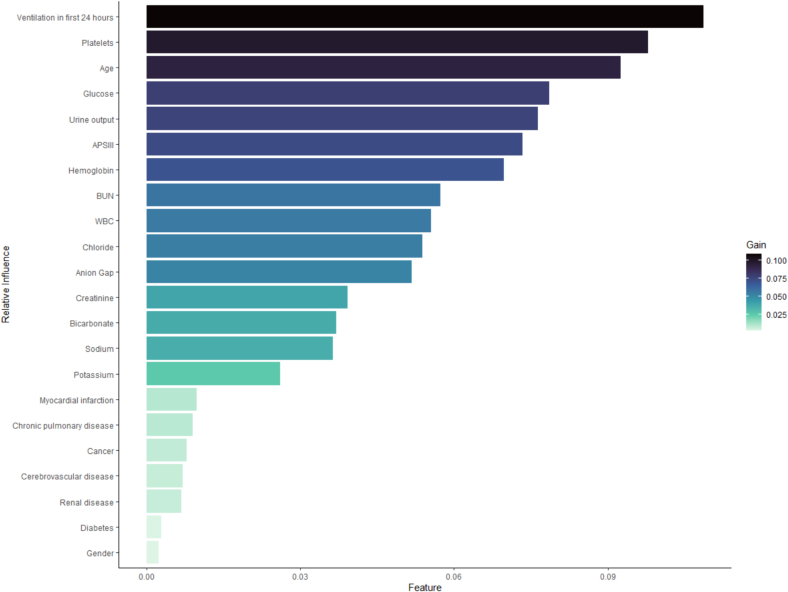
Relative influence of characteristics predicting the likelihood of a patient having an indwelling arterial catheter in the ICU.

The propensity scores for each individual were calculated using an XGboost model incorporating all the features list in Table 1 (details on the matching effects are presented in Fig. S1 in the Supplementary Material). The importance of these features in the model is illustrated in Fig. 2. Activation of mechanical ventilation within the first 24 h and oxygen saturation were identified as the two most important features in determining the propensity scores for intra-arterial catheters. This finding aligns with clinical practice, as patients on mechanical ventilation often require frequent arterial blood gas sampling, thus justifying the use of indwelling arterial catheters for convenience.

Doubly robust estimation confirmed the lack of significant difference in 28-day mortality between the two patient groups. Sensitivity analyses, employing the propensity-matched allocation method indicated that the mortality rates in the arterial catheter and non-arterial catheter groups were 7.14 % and 10.5 %, respectively (odds ratio [OR]: 0.67, 95 % confidence interval [CI]: 0.35–1.25, *P* = 0.268) (details on propensity score matching are presented in Table S2 in the Supplementary Material). The specific results of the primary outcomes are summarized in Table 2.

**Table 2:** Primary outcome analysis.

Methods	OR	95%CI	*p*
Doubly robust with unbalanced covariates	0.61	0.31–1.21	0.155
Doubly robust with all covariates	0.64	0.31–1.32	0.225
Propensity score IPW	0.61	0.31–1.19	0.147
Propensity score matching	0.67	0.35–1.25	0.268

OR: Odds ratio, CI: Confidence Interval.

**Table 1:** Demographics of the cohort.

Covariate	Non-arterial catheters group	Arterial catheters group	*p*
Number	1722	347	
Male(%)	886 (51.5)	187 (53.9)	0.441
Age (median [IQR])	76.69 [65.22, 85.56]	74.08 [64.80, 83.30]	0.018
Hospital mortality(%)	107 (6.2)	9 (2.6)	0.011
28-day mortality(%)	215 (12.5)	19 (5.5)	<0.001
90-day mortality(%)	358 (20.8)	37 (10.7)	<0.001
Length of hospital stay (day) (median [IQR])	7.75 [5.01, 11.96]	9.96 [6.90, 14.30]	<0.001
ICU free days within 28 days (day) (median [IQR])	25.60 [24.14, 26.36]	24.63 [22.66, 25.98]	<0.001
Urine output in first 24 h (ml/kg) (median [IQR])	24.02 [14.76, 36.29]	20.17 [13.31, 32.93]	0.011
Laboratory results			
Hemoglobin (g/dL) (median [IQR])	10.85 [9.30, 12.45]	10.75 [9.40, 12.15]	0.695
WBC (*10^9^/L) (median [IQR])	9.70 [7.40, 12.89]	10.20 [8.10, 13.47]	0.014
Platelets (*10^9^/L) (median [IQR])	210.25 [162.12, 273.00]	194.50 [152.25, 252.50]	0.001
Glucose(mg/dL) (median [IQR])	132.67 [111.75, 169.92]	133.00 [117.00, 161.12]	0.732
Anion gap (mmol/L) (median [IQR])	15.00 [13.00, 17.50]	14.50 [12.50, 16.00]	<0.001
Bicarbonate (mmol/L) (median [IQR])	25.00 [22.00, 28.00]	24.50 [22.00, 27.50]	0.145
BUN (mg/dL) (median [IQR])	29.00 [19.50, 46.38]	25.00 [18.00, 37.00]	<0.001
Chloride (mmol/L) (median [IQR])	101.00 [97.00, 104.50]	102.50 [99.00, 106.00]	<0.001
Creatinine (mmol/L)(median [IQR])	1.30 [0.95, 1.95]	1.20 [0.90, 1.70]	0.031
Sodium (mmol/L)(median [IQR])	138.50 [135.50, 141.00]	138.50 [136.50, 141.00]	0.33
Potassium (mmol/L)(median [IQR])	4.25 [3.85, 4.65]	4.15 [3.85, 4.55]	0.049
Treatment in first 24 h			
Mechanical ventilation (%)	219 (12.7)	132 (38.0)	<0.001
Illness severity score			
APSIII (median [IQR])	42.00 [33.00, 51.00]	43.00 [34.50, 54.00]	0.055
Comorbidity			
Myocardial infarction (%)	612 (35.5)	104 (30.0)	0.054
Cerebrovascular disease (%)	150 (8.7)	48 (13.8)	0.004
Chronic pulmonary disease (%)	698 (40.5)	132 (38.0)	0.421
Renal disease (%)	740 (43.0)	136 (39.2)	0.215
Diabetes (%)	731 (42.5)	144 (41.5)	0.789
Cancer (%)	192 (11.1)	23 (6.6)	0.015

APSIII Acute Physiology Score III; BUN Blood urea nitrogen; IQR interquartile range; WBC White blood cell.

### 3.3 Secondary outcomes

Secondary outcome analysis revealed a lack of significant difference in 90-day mortality between the arterial catheter group and the non-arterial catheter group (OR: 0.67, 95 % CI: 0.42–1.08, *P* = 0.104). However, a lower rate of in-hospital mortality was observed in the arterial catheter group than that in non-arterial catheter group (OR: 0.41, 95 % CI: 0.14–0.65, *P* = 0.02). Analysis regarding ICU free days within 28 days demonstrated that the difference between patients with and without arterial catheter placement was not statistically significant (estimated β: -0.285, 95 % CI: −0.985 to 0.4149, *P* = 0.425).

## 4 Discussion

Our findings did not demonstrate a significant association between intra-arterial catheterization and 28- or 90-day mortality or a variance in 28-day ICU free days. However, a reduction in in-hospital mortality was noted in patients with acute heart failure without shock. While these findings provide valuable insights, the retrospective nature of the study implies that they represent correlations, not causation. Despite adjusting for confounders using propensity score weighting and logistic regression, unknown variables may still influence the outcomes. Thus, our results should be viewed as preliminary, establishing groundwork for future research to definitively ascertain the effects of intra-arterial catheterization in such patients.

Given these limitations and findings, several explanations can account for these observations. First, intra-arterial catheterization in the ICU facilitates hemodynamic monitoring and influences treatment decisions. However, factors beyond the hospital may outweigh the benefits of the arterial placement, impacting long-term mortality outcomes. Rosa et al. have consistently identified ICU-acquired infections as the primary contributor to increased early mortality among patients discharged from the ICU (20). Arterial catheters are associated with bloodstream infections (21); however their association with early post-discharge infections remains uncertain. Second, potential thrombosis complications associated with arterial catheter placement, which can manifest up to 75 days after catheter placement, must be considered (22). Thrombotic events contribute to readmission rates within 90 d in patients with heart failure, with a reported rate of 3.9 %, and have been associated with increased mortality in these patients following discharge (23). Third, despite collecting data on over 30 covariates, certain confounding factors that could influence the association between arterial catheters and 30- and 90-day mortality could have been overlooked.

The present study has several limitations. First, we did not differentiate patients with arterial catheters by placement site, which influences the propensity for infection (21). However, information on the catheters' location is missing for some entries in the database, and the exclusion of such entries would have further reduced the sample size. Second, the characteristics we collected were all data from within 24 h of transferring the patient to the ICU, which could not fully reflect the dynamic course of the patient in the ICU. Third, this was a single-center retrospective study with a large time span. Some potential confounding factors, such as changes in treatment strategy, were not considered. Furthermore, while we utilized advanced statistical techniques such as XGBoost and doubly robust estimation to minimize bias and enhance the robustness of our findings, these methods cannot eliminate all biases inherent in retrospective studies. Additionally, we could not distinguish between HFrEF and HFpEF due to the lack of echocardiographic data, and we lacked data on NT-pro-BNP because many patients, especially those from earlier years in the database, did not undergo this test. Finally, this study design cannot account for causality, and further randomized controlled trials are required to validate our findings.

## 5 Conclusion

Our single-center retrospective analysis indicates that arterial catheterization in acute heart failure patients without shock is not associated with changes in 28- or 90-day mortality or 28-day ICU-free days. However, it is linked to a decrease in in-hospital mortality. These results emphasize the complex impact of arterial catheterization in this patient cohort.

## List of abbreviations

BIDMC: Beth Israel Deaconess Medical Center
CI: Confidence Interval
HFrEF: Heart Failure with Reduced Ejection Fraction
HFpEF: Heart Failure with Preserved Ejection Fraction
ICD: Intra-arterial Catheters
ICU: Intensive Care Unit
IPW: Inverse Probability Weighting
MIMIC-IV: Medical Information Mart for Intensive Care-IV
NT-pro-BNP: N-terminal pro-B-type natriuretic peptide
OR: Odds Ratio
SD: Standard Deviation
XGBoost: eXtreme Gradient Boosting+

## Consent for publication

The manuscript does not contain details, images, or videos relating to an individual person. Therefore, consent for publication is not required.

## Funding

This research received no specific grant from any funding agency in the public, commercial or not-for-profit sectors.

## Ethic statement

The need for patient consent was waived owing to the retrospective nature of this study based on deidentified dataset. The Institutional Review Boards at both Massachusetts Institute of Technology (Protocol No. 0403000206) and the Beth Israel Deaconess Medical Center (BIDMC) (Protocol No. 2001-P-001699/14) approved the use of the data for research.

## CRediT authorship contribution statement

**Yide Li:** Writing – review & editing, Resources, Formal analysis. **Yuan Zhu:** Writing – original draft. **Le Fu:** Validation. **Liang Luo:** Supervision, Project administration, Conceptualization. **Yingfang She:** Supervision, Project administration, Conceptualization.

## Declaration of competing interest

The authors declare that they have no known competing financial interests or personal relationships that could have appeared to influence the work reported in this paper.

## Data Availability

Due to the licensing restrictions of the MIMIC database, we are unable to provide the data file directly. However, the source code for all analyses conducted in this study can be accessed at https://github.com/shaou77/IACinAHF upon the publication of this paper. Alternatively, you may contact the corresponding author, Liang Luo, via email at luoliang@mail.sysu.edu.cn for further inquiries.

## Appendix A Supplementary data

Supplementary material Image 1
