# Supplementary material for: Association between intra-arterial catheterization and mortality of acute heart failure patients without shock in ICU: A retrospective study
Source: Am Heart J Plus. 2024 Jul 29;45:100432. doi: 10.1016/j.ahjo.2024.100432 (PMC11345900; doi:10.1016/j.ahjo.2024.100432)

Supplementary Material

Association between Intra-arterial Catheterization and Mortality of Acute Heart Failure Patients without Shock in ICU: A Retrospective Study

**Supplementary Table 1. The ICD code for ‘acute heart failure’ diagnosis**

| ICD code | ICD version | Diagnosis name |
| --- | --- | --- |
| 42821 | 9 | Acute systolic heart failure |
| 42823 | 9 | Acute on chronic systolic heart failure |
| 42831 | 9 | Acute diastolic heart failure |
| 42833 | 9 | Acute on chronic diastolic heart failure |
| 42841 | 9 | Acute combined systolic and diastolic heart failure |
| 42843 | 9 | Acute on chronic combined systolic and diastolic heart failure |
| I5021 | 10 | Acute systolic (congestive) heart failure |
| I5023 | 10 | Acute on chronic systolic (congestive) heart failure |
| I5031 | 10 | Acute diastolic (congestive) heart failure |
| I5033 | 10 | Acute on chronic diastolic (congestive) heart failure |
| I5041 | 10 | Acute combined systolic (congestive) and diastolic (congestive) heart failure |
| I5043 | 10 | Acute on chronic combined systolic (congestive) and diastolic (congestive) heart failure |
| I50811 | 10 | Acute right heart failure |
| I50813 | 10 | Acute on chronic right heart failure |

**Supplementary Table 2 Comparison of the covariate of Unmatched Cohorts and Propensity-Matched Cohorts in Sensitivity Analysis**

| Covariate | Original cohort | | | Matched cohort | | |
| --- | --- | --- | --- | --- | --- | --- |
|  | Non-arterial catheters group | Arterial catheters group | *p* | Non-arterial catheters group | Arterial catheters group | *p* |
| Number | 1722 | 347 |  | 238 | 238 |  |
| Male(%) | 886 (51.5) | 187 (53.9) | 0.441 | 127 (53.4) | 129 (54.2) | 0.927 |
| Age (median [IQR]) | 76.69 [65.22, 85.56] | 74.08 [64.80, 83.30] | 0.018 | 72.00 (14.65) | 73.40 (15.19) | 0.306 |
| Urine output in first 24 h (ml/kg) (median [IQR]) | 24.02 [14.76, 36.29] | 20.17 [13.31, 32.93] | 0.011 | 26.34 (18.36) | 27.56 (19.08) | 0.478 |
| Laboratory results |  |  |  |  |  |  |
| Hemoglobin (g/dL) (median [IQR]) | 10.85 [9.30, 12.45] | 10.75 [9.40, 12.15] | 0.695 | 10.97 (2.13) | 10.95 (2.11) | 0.891 |
| WBC (*109/L) (median [IQR]) | 9.70 [7.40, 12.89] | 10.20 [8.10, 13.47] | 0.014 | 11.13 (5.85) | 10.99 (7.79) | 0.819 |
| Platelets (*109/L) (median [IQR]) | 210.25 [162.12, 273.00] | 194.50 [152.25, 252.50] | 0.001 | 203.53 (82.79) | 209.30 (88.16) | 0.462 |
| Glucose(mg/dL) (median [IQR]) | 132.67 [111.75, 169.92] | 133.00 [117.00, 161.12] | 0.732 | 146.42 (46.51) | 144.77 (42.86) | 0.687 |
| Anion gap (mmol/L) (median [IQR]) | 15.00 [13.00, 17.50] | 14.50 [12.50, 16.00] | <0.001 | 14.58 (3.25) | 14.82 (3.07) | 0.404 |
| Bicarbonate (mmol/L) (median [IQR]) | 25.00 [22.00, 28.00] | 24.50 [22.00, 27.50] | 0.145 | 25.26 (5.00) | 25.27 (4.91) | 0.989 |
| BUN (mg/dL) (median [IQR]) | 29.00 [19.50, 46.38] | 25.00 [18.00, 37.00] | <0.001 | 32.01 (20.79) | 33.04 (22.12) | 0.6 |
| Chloride (mmol/L) (median [IQR]) | 101.00 [97.00, 104.50] | 102.50 [99.00, 106.00] | <0.001 | 102.00 (5.77) | 101.45 (5.66) | 0.3 |
| Creatinine (mmol/L)(median [IQR]) | 1.30 [0.95, 1.95] | 1.20 [0.90, 1.70] | 0.031 | 1.54 (1.17) | 1.51 (1.10) | 0.778 |
| Sodium (mmol/L)(median [IQR]) | 138.50 [135.50, 141.00] | 138.50 [136.50, 141.00] | 0.33 | 138.54 (4.07) | 138.15 (4.21) | 0.296 |
| Potassium (mmol/L)(median [IQR]) | 4.25 [3.85, 4.65] | 4.15 [3.85, 4.55] | 0.049 | 4.23 (0.58) | 4.18 (0.53) | 0.361 |
| Treatment in first 24 h |  |  |  |  |  |  |
| Mechanical ventilation (%) | 219 (12.7) | 132 (38.0) | <0.001 | 98 (41.2) | 45 (18.9) | <0.001 |
| Illness severity score |  |  |  |  |  |  |
| APSIII (median [IQR]) | 42.00 [33.00, 51.00] | 43.00 [34.50, 54.00] | 0.055 | 44.55 (14.99) | 43.18 (14.46) | 0.311 |
| Comorbidity |  |  |  |  |  |  |
| Myocardial infarction (%) | 612 (35.5) | 104 (30.0) | 0.054 | 71 (29.8) | 72 (30.3) | 1 |
| Cerebrovascular disease (%) | 150 (8.7) | 48 (13.8) | 0.004 | 28 (11.8) | 30 (12.6) | 0.889 |
| Chronic pulmonary disease (%) | 698 (40.5) | 132 (38.0) | 0.421 | 100 (42.0) | 88 (37.0) | 0.302 |
| Renal disease (%) | 740 (43.0) | 136 (39.2) | 0.215 | 88 (37.0) | 91 (38.2) | 0.85 |
| Diabetes (%) | 731 (42.5) | 144 (41.5) | 0.789 | 108 (45.4) | 94 (39.5) | 0.228 |
| Cancer (%) | 192 (11.1) | 23 (6.6) | 0.015 | 10 (4.2) | 13 (5.5) | 0.669 |

**Supplementary Figure 1 Graphical Evaluation of XGBoost Model Balance**

"ks.mean": The average Kolmogorov-Smirnov statistic stopping criterion.

"ks.max": The maximum Kolmogorov-Smirnov statistic stopping criterion.

"es.mean": The average standardized effect size stopping criterion.

"es.max": The maximum standardized effect size stopping criterion.

These four statistics are used to assess the balance and magnitude of differences between the groups, helping to evaluate the effectiveness of the estimation process and the sensitivity of the results to different model stopping rules.

A. Effect Size Plot of Weighted Differences

Blue lines: Magnitudes of differences between groups on each pretreatment covariate after applying weights. The magnitudes are standardized using the standardized effect size.

Red lines: Magnitudes of differences between groups on each pretreatment covariate after applying weights. Only several covariates shows an increase in effect size, which is seemingly trivial.

Closed red circles: Indicate statistically significant differences. These occur before weighting, but few after weighting.


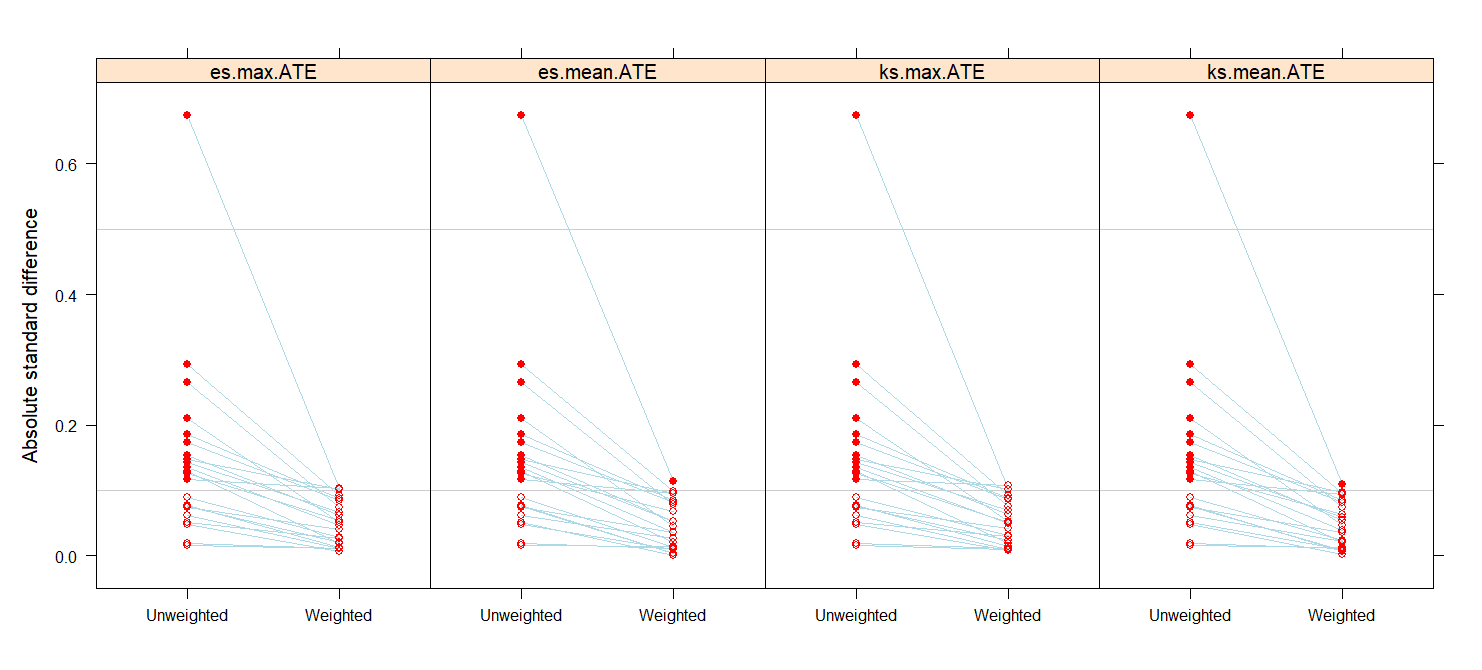


B. Assessment of Balance Using QQ Plot of P-Values for Covariate Differences

In the QQ plot, p-values below the diagonal indicate lack of balance, while p-values running at or above the diagonal suggest balance may have been achieved. The plot compares the observed p-values to the quantiles of the uniform distribution, allowing users to visually inspect the p-values of KS statistic for group differences in covariate means.


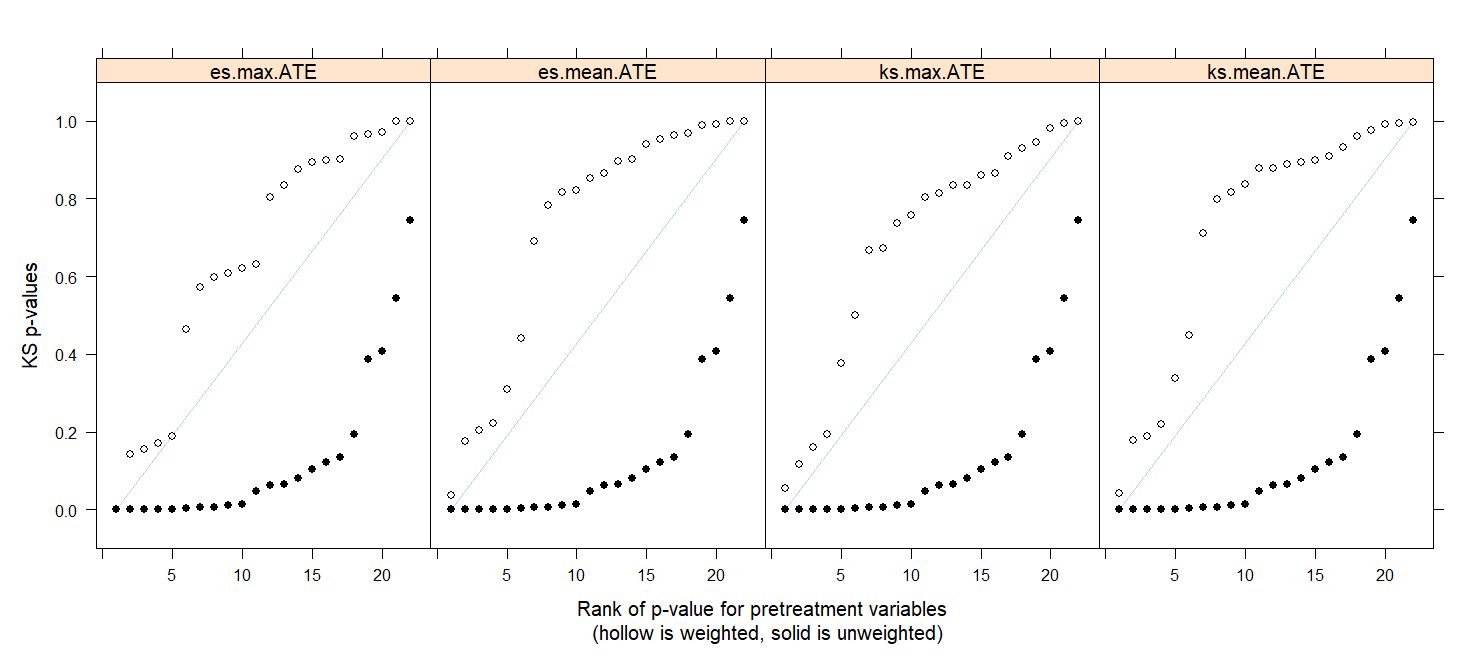

Supplement: Supplementary file 1 — Supplementary material [file mmc1.docx]
